# *Mycobacterium Avium Paratuberculosis*: A Disease Burden on the Dairy Industry

**DOI:** 10.3390/ani10101773

**Published:** 2020-10-01

**Authors:** Mary Garvey

**Affiliations:** Department of Life Science, Sligo Institute of Technology, F91 YW50 Sligo, Ireland; garvey.mary@itsligo.ie; Tel.: +353-071-9305529

**Keywords:** infectious disease, Mycobacterium, economic impact, prevalence, control

## Abstract

**Simple Summary:**

The causative agent of Johne’s disease is *Mycobacterium avium* *paratuberculosis*, a virulent contagious bacterial species. At present, Johne’s disease is an endemic disease of beef and dairy farming. This disease incurs serious economic costs and creates animal welfare issues in food production. Disease management is often ineffective due to poor diagnostic techniques, subclinical infections, high fecal shedding, vertical transmission, and lack of producer awareness. Furthermore, foodborne transmission and a correlation with human inflammatory bowel disease further strengthen the importance of this pathogen. Harmonized effective biosecurity measures and timely culling of high-shedding animals are essential in limiting the spread of disease. At farm level, this is not always implemented, however, as producers often do not recognize the importance of Johne’s disease at production level. To improve animal welfare and ensure public health safety, it is imperative to better understand the factors promoting *Mycobacterium avium* pathogenicity and virulence.

**Abstract:**

**Mycobacterium avium* paratuberculosis* is responsible for paratuberculosis or Johne’s disease in cows, having economic impacts on the dairy industry and a prevalence rate exceeding 50% in dairy herds. The economic burden of Johne’s disease relates to decreased milk production and costs of disease prevention, treatment, and management, while having an economic impact on dairy producers, processors, consumers, and stakeholders of the dairy industry. Determining the true economic impact of the disease is difficult at regional and farm level as symptoms are not evident in subclinically infected animals. At present, the virulence, pathogenicity, persistence, and infectious dose of *M. avium paratuberculosis* are poorly understood, consequently effective paratuberculosis control measures remain obscure. *M. avium paratuberculosis* is potentially zoonotic with foodborne transmission a public health risk due to a possible causative link with inflammatory bowel disease in humans. A preventive approach is necessary to reduce the presence of this drug-resistant pathogen in dairy herds and subsequently dairy food. The use of inefficient diagnostic tests coupled with the long latency period of infection results in delayed animal culling and trade of asymptomatic animals, leading to regional transmission and increased disease prevalence. To date, there has been limited success at controlling and treating this terminal endemic disease, leading to significant prevalence rates. This study aims to outline the key factors associated with Johne’s’ disease while outlining its significant impact on the dairy sector.

## 1. Introduction

The dairy industry is a vital source of nutrient-dense food and employment globally as it represents a nutritious food source, essential in both developed and developing countries. Infectious diseases occur endemically and epidemically in dairy operations globally. Dairy production is impacted by cow and herd health with infectious disease having drastic effects on milk yield, milk quality, cow well-being, and the economic welfare of the producer. Infectious disease also promotes the oral administration of antibiotics to cattle which is a leading factor for the development of antimicrobial resistance (AMR) in enteric pathogens. The prevention and control of infectious disease and AMR are now priority animal health initiatives of the One Health approach [1]. One Health is an important initiative established to reduce and limit the occurrence of infectious disease in animal and human populations. A microbial disease impacting the dairy industry is Johne’s disease (JD) or paratuberculosis, which is resultant from intestinal infection with the pathogenic species **Mycobacterium avium* paratuberculosis* (MAP), a Gram-positive acid-fast bacterium and a member of the *M. avium* complex group [2]. JD affects many ruminant species both wild and domestic including sheep, goat, deer, camelids, and primates [3], causing a chronic, progressive, contagious infection. The production impact of JD is due to reduced milk yield, cow culling, reduced calving rates, infertility, disease treatment, and control costs. Subclinical cases of infection do not manifest with symptoms and often persist undetected, causing a prolonged reduction in milk yield. Clinical JD manifests with diarrhea, decreased milk production, edema, infertility, and weight loss and is controlled by animal culling [4] to prevent disease transmission. Furthermore, MAP may be zoonotic in nature with a speculative causative link to Crohn’s disease (CD) in humans, an autoimmune disease associated with chronic wasting of the human intestines [5]. Indeed, there is a possible link between MAP and numerous other autoimmune diseases such as type 1 diabetes, rheumatoid arthritis, and multiple sclerosis (MS) amongst others [5,6]. This link has been correlated by the pathophysiological similarities of JD and CD coupled with the detection of MAP in dairy food produce and in the intestinal tract of Crohn’s patients [7]. Raw milk is a route of human exposure to MAP; however, studies have shown that MAP is capable of surviving pasteurization techniques, making processed milk also a possible route of transmission [8]. MAP has been detected in the milk of both clinical and subclinically infected cows and is estimated to occur in 1 to 3% of milk globally, leading the European Union to implement MAP control programs [9] to ensure public health safety. The detection of MAP in infant milk powder is a major concern particularly in relation to Crohn’s disease in pediatric cases [10]. The excretion of MAP-laden feces by cows leads to contamination of surface waters from agricultural run-off, which can then enter drinking water supplies, another route of transmission.

MAP poses considerable challenges relating to disease control due to a lack of reliable diagnostic tests and its intrinsic resistance to antibiotics and disinfectants. The potential causative relationship between MAP and numerous autoimmune conditions [6] in humans, the economic burden MAP infection puts on the dairy industry, and associated animal welfare issues make MAP an ongoing global issue. Understanding the etiology of disease, bacterial transmission, and effective control measures is vital in controlling this pathogen at a national and international level. This review aims to inform on aspects of MAP pathogenicity by providing an insight into the factors associated with MAP infectious disease.

## 2. The Triad of Johne’s Disease

**Figure 1:**
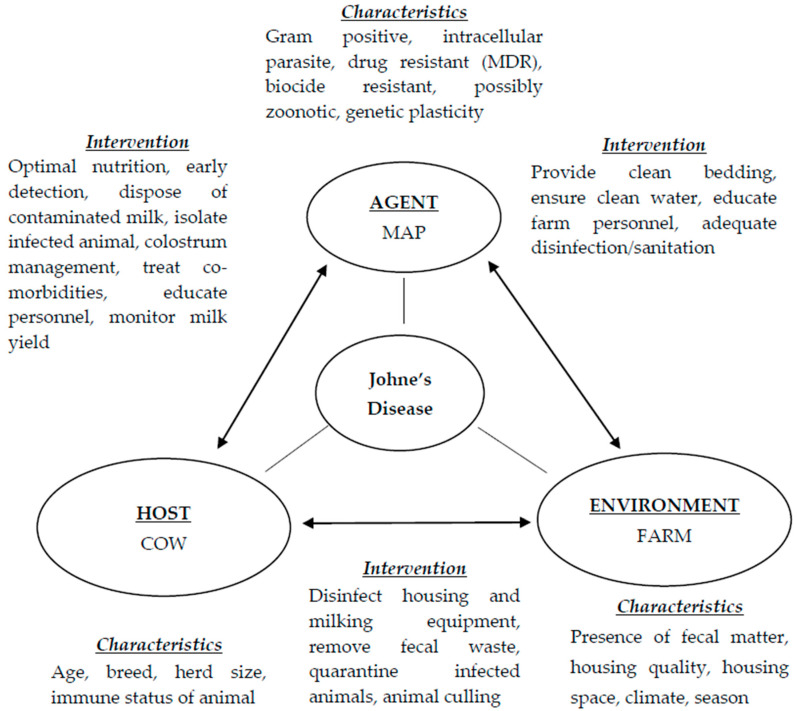
The triad of Johne’s disease outlining associated characteristics and intervention methods for disease prevention on dairy farms.

The triad of disease is an epidemiological concept used to gain insight into the spread of infectious disease endemically and pandemically. Infectious disease is a result of the interplay between the host and pathogen involving the host defense mechanisms (immune system) and the pathogen’s efficiency at evading the host immune response [11]. The triad consists of the agent (pathogen), the host (infected animal), and the environment (area where patient resides). In terms of Johne’s disease, the agent is MAP, the host is the cow, and the environment refers to the housing area/farming environment. The interaction between these three factors therefore (Figure 1) enables bacterial colonization of the host and disease transmission within herds and possibly, the external animal host range. Johne’s disease is described in stages where stage 1 is considered a silent stage often present in young calves and heifers which is undetectable. Stage 2 is subclinical asymptomatic infection in adults (2 to 5 years) [12] and can be detected by antibody tests. Stage 3 prolonged clinical disease manifests with the symptoms of disease including reduced milk yield and histopathological lesions with stage 4 associated with disseminated infection, severe dehydration, cachexia, and death [12].

### 2.1. The Pathogen—MAP

**Mycobacterium avium* paratuberculosis* is a member of the *Mycobacterium avium* complex (MAC) in the genus Mycobacterium and family *Mycobacteriaceae* [13]. The MAC is a group of related species of nontuberculosis *mycobacteria*, the most isolated species in human infections such as pulmonary disease and dermal, soft tissue, and systemic infections [14]. MAC consists of two distinctly classified species, namely *M. avium* and *M intracellulare*. There are four subspecies of *M. avium* as segregated by 16S-23S ribosomal RNA sequencing into *M. avium avium*, *M. avium hominissuis* (MAH), MAP, and *M. avium. silvaticum* [13]. MAP is an obligate intracellular parasite due to its lack of mycobactin (an iron-chelating compound) and subsequent need for a source of iron. MAP has a slow reproduction rate with a doubling time exceeding 22–26 h [11]. As a Gram-positive, rod-shaped, acid-fast bacterium, MAP has a thick waxy cell wall made up of mycolate and peptidoglycan layers held together by arabinogalactan [15]. This cell wall structure allows the pathogen to survive in the environment outside of the ruminant host, such as feces, and possibly animal food products [16]. In the environment, MAP may be found as a cell wall-containing form, in a dormant vegetative state, or as a heat-resistant spore-like form [17]. Within a host, the cell wall is typically absent, providing resistance to numerous antibiotic drugs, namely therapeutics targeting peptidoglycan biosynthesis including penicillin, cephalosporin, and vancomycin [5], making MAP intrinsically multidrug-resistant (MDR).

As the etiology of disease, MAP can alter its phenotype throughout the various stages of disease depending on the environment (epithelial cells of mammary glands, milk), indicating genetic plasticity. The upregulation of certain MAP genes (Table 1) in a hyperosmolar milk environment, in particular, results in the formation of more virulent and infectious phenotypes [18]. Such an environment is believed to trigger the expression of cellular invasion virulence factors seen in many species including Salmonella, Shigella, *E. coli* [19], and MAP [20]. To gain entry to the systemic circulation, MAP is effective at binding and crossing the mucosal barrier in ruminants where it can transverse the mucosa asymptomatically [3]. To survive within the host macrophage, MAP prevents cell apoptosis, evades phagolysosome activity, avoids detection via altering chemokine and cytokine signaling, and alters dendritic cell maturation [21]. *Mycobacterium avium* species are also capable of resisting reactive oxygen intermediates and nitric oxide [22], both potent antibacterial compounds [23].

### 2.2. The Host

In the dairy herd, MAP is transmitted horizontally primarily by the fecal–oral route and vertically in utero, in the milk and colostrum (as determined via PCR) to newborn calves. The colostrum is an important source of growth factors, vitamins, and immunoglobulins (IgGs) as calves have agammaglobulinemia at birth [24] and require these IgGs via colostrum. As such, animals become infected within the first month of life with bovine susceptibility to MAP infection decreasing with age [25]. The innate immune response of the animal is the primary frontline defense system, namely the mucosal intestinal lining, macrophages, dendritic cells, epithelial tight junctions, and antimicrobial peptides of the Peyer’s patches [26]. Bovine macrophages, however, become the residence of this intracellular parasite as MAP has developed mechanisms allowing it to colonize and reproduce within these normally phagocytotic cells [4]. Indeed, in dairy cows, MAP infection occurs largely in young ruminants when calves are more susceptible to infection due to the increased presence of transient ileal Peyer’s patches in young ruminants [21]. Peyer’s patches provide many microfold cells (M cells) allowing for the uptake of MAP. M cells also act as a gateway of entry for other enteropathogenic bacteria, including *E. coli*, *Vibrio cholerae*, *Salmonella*, and *Shigella* species [26]. The movement of MAP through the ruminant gut stimulates the bacterial cell wall protein fibronectin attachment protein (FAP), which promotes opsonization by fibronectin [25,26] subsequently connecting MAP to the luminal surface of these M cells via attachment to fibronectin receptors [27], allowing for MAP uptake. MAP encounters dendritic cells and subepithelial macrophages in the lamina propria (a layer of loose connective tissue) after movement across the intestinal epithelial layer. After infection is established, MAP colonizes the macrophages and dendritic cells in the distal ileum of the bovine intestines, where it modulates the intracellular environment, cell surface molecules, and cytokine release to maintain a prolonged intracellular presence [25]. The anti-inflammatory cytokine IL-10 represses inflammatory immune responses allowing the growth of MAP within macrophages [28]. Host compounds appear to stimulate MAP invasion and colonization [26]. Studies have identified several MAP genes within the host which are cofactors at the different stages of infection [29]. The presence of infection inducing MAP antibodies may also enhance the uptake of MAP. Indeed, maternal MAP antibodies given to calves via the colostrum may promote uptake via increased opsonization of the bacteria [3]. Once infection of the host has occurred, there is a prolonged asymptomatic latency period of three to five years [17], often accompanied with fecal shedding. The adaptive immune system and specific antibody response is stimulated with the circulation of large numbers of extracellular MAP in the bloodstream [30] after crossing the intestinal epithelium. Fecal shedding is more prominent during this adaptive immune response and clinical stage of disease. Once MAP has successfully colonized the ruminant gut, the tissues develop a characteristic granuloma, histological, and gross appearance, leading ultimately to a lack of nutrient absorption [31] and malnutrition in the animal. The characteristic signs of advanced stage disease in cattle is copious diarrhea and visual appearance of the ribs and other skeletal bones [32]. Disseminated or systemic infections were thought to occur when the disease was at this advanced stage. More recent studies suggest, however, that in cases of young animals, episodic disseminated infections can occur during the earlier stages of disease [31].

Infection with MAP does not necessarily result in Johne’s disease; additional factors including parturition, lactation, age, herd size, and immunity are cofactors for the manifestation of clinical disease. Notably, despite the virulence of the MAP pathogen, approximately 10–15% of exposed cattle develop clinical disease, demonstrating an ability of calves to fight and eliminate the infection [33]. Host genetic factors are believed to play a role in this resistance to infection; these factors, however, are extremely difficult to analyze and determine [25]. Factors include host health and immunity, environmental conditions, the infectious dose (currently undetermined), and variables associated with varying MAP phenotypes. There is an urgent need for specifics on such predictors to establish preventative control measures. Animal trade and movement effectively spread JD to new regions. MAP has been identified in bovine semen and reproductive organs, suggesting breeding stock may also serve as possible routes of disease transmission [34].

**Table 1:** Mycobacterial virulence factors promoting infection and disease.

Virulence Factor	Function	Disease Promotion
Upregulated genes * MAP1203	Invasion and intracellular persistence protein	Cellular invasion within the host, avoidance of host immunity
MAP4088	Cell entry lipoprotein	
LuxR [29]	Gene overexpression increases cellular invasion [18]	
Nonpolar lipid (lipid 550) [26]	Cell wall lipid	
Downregulated gene * para-LP-01 [26]	Cell wall component	Downregulated in vivo promoting AMR
Absence of cell wall in vivo	Intracellular parasite, reduced genome size	Resistance to antibiotic therapy, difficult to detect
Genetic plasticity	Production of varying phenotypes	Adaption to environmental changes, promotes survival
Mucosal adherence	Colonization of host membranes	Avoidance of host immunity, access to macrophages
Fibronectin attachment protein	Attachment and internalization of *mycobacteria* [35]	Promotes opsonization by fibronectin [27]
Altering phagolysosome fusion and maturation [25]	Disrupt the formation of the mature phagolysosome, prevent acidification	Survival within macrophages, avoid hydrolysis and oxidation reactions [25]
Modulation of macrophage apoptosis	Controlling apoptosis, regulating macrophage cell death	Allows for intracellular MAP replication [36] until burst capacity is reached [21]
PstA gene [37]	Biofilm formation-populated communities attached to surfaces	Chemical resistance, surviving harsh environments
Dormancy	Bacterial state allowing survival of non-spore-forming bacteria [38,39]	Survival in unfavorable environmental conditions [38]
Heat tolerance [28]	Resistance to pasteurization	Foodborne zoonosis

* strain specific.

### 2.3. The Environment

The farm environment acts as a natural reservoir for numerous pathogenic species with transmission within herds via water and fecal matter extremely likely. Furthermore, dairy cattle are often reservoirs for many zoonotic pathogens (*E. coli*, *Salmonella*, *Cryptosporidium*) leading to foodborne disease. The relationship between MAP and the environment is complicated, dependent on many factors including the physical characteristics of its location such as feces, water, milk, manure, dust, fomites, temperature, pH, water content, and rival microorganisms. The shedding of MAP in feces in subclinical and clinically infected cows is a major source of environmental MAP, with water contamination from land run-off a concern. Once excreted, MAP can survive outside the host for 12 weeks and up to 120 weeks in soil or water [40]. Internalization within free-living amoeba promotes the survival of MAP and other pathogens (*Legionella*, *Listeria*, *Campylobacter jejuni*, *Helicobacter pylori*) in aquatic environments [41] and promotes chlorine resistance [28]. MAP also demonstrates resistance to high temperatures and low pH, where humidity and UV light can reduce the environmental survival time of the pathogen [40]. The lipid-rich cell wall present in environmental MAP promotes survival in adverse conditions. Whether dairy cows are housed indoors or free to graze affects direct and indirect MAP transmission. The structure and management of herd housing also influences infectious disease transmission. Calf-to-calf transmission occurs from contact to contaminated housing surfaces (indirect transmission) whereas adult-to-calf transmission is dependent on calves being exposed to feces on housing fomites [42]. Grazing on fecal-contaminated pastures and waterways allows for environmental exposure in herds not housed. Aerosol transmission is also possible as viable MAP has been recovered from air samples and water droplets [43]. MAP-contaminated aerosols could potentially travel large distances depending on wind and deposit on land or water, subsequently being ingested or inhaled by grazing animals [39]. *Mycobacteria* are competent biofilm producers and can attach and grow on environmental surfaces, producing a persistent source of infection, possessing chemical resistance [37].

## 3. Prevalence of Disease

Studies show a prevalence of above 50% for MAP in dairy herds in countries operating intensive farming systems [40] with low levels (5%) of clinical disease [44]. Indeed, in the United States, prevalence ranges from 68 to 91% in dairy herds and 8% of beef herds [45] with estimates typically based on ELISA, fecal culture, or PCR techniques. Estimating prevalence of infection is difficult as identification of MAP is achieved mostly via fecal culture, with no symptoms of infection in the animal subclinically. The clinical stage manifests with high levels of fecal shedding, chronic diarrhea, rapid weight loss, decreased milk production, edema, infertility, and death [46]. At this terminal stage, the animal is typically culled and transmission within the herd may have already occurred. MAP has a long incubation period prior to the manifestation of Johne’s disease, making early detection difficult, as seen with many endemic diseases [47]. In the clinical stage of disease, an animal may transmit to adjacent animals, depending on factors such as close contact and animal husbandry [28]. Diagnostic tools available for detection differ in their sensitivity and specificity, confounding data on herd prevalence. For example, the application of ELISA tests to detect MAP antibodies is confounded by common antigens between other Mycobacterium species such as M. tuberculosis and other MAC strains, reducing specificity [44]. The ELISA test detects specific MAP antibodies and is based on a humoral immune response in the animal, typically seen in late-stage clinical disease [48], rendering it ineffective for subclinical infections. Current ELISA tests, however, have a specificity of 98% (clinical disease) and are less expensive and user friendly, with a shorter turnaround time than culture or PCR assays [46].Quantitative real-time PCR (qPCR) has demonstrated greater sensitivity and specificity (60 and 97%, respectively) for the detection of MAP in milk, fecal, and environmental samples [49]. IS900 is the molecular target for PCR amplification, however, this sequence can be detected in other Mycobacterium species, leading to false positives. Cultural isolation of MAP is considered the gold standard for detection of the pathogen with a sensitivity of less than 30% and specificity of 100% in subclinically infected cattle [24]. The culture of fecal MAP is dependent on the stage of disease as the pathogen is excreted intermittently and to varying extents depending on the animal. Determination of true prevalence levels is difficult due to the variations in accuracy of diagnostic tests, MAP strain variations, bacterial cultural requirements, and the nature of the disease [50]. To establish accurate prevalence endemically and among countries where trade is common, more accurate diagnostic tests are required to identify the pathogen at the early stages of infection.

## 4. Impact on the Dairy Industry

Estimates show global economic losses exceeding 1.5 billion US dollars annually from endemic cases of Johne’s disease [32]. Economic losses in dairy industries across the globe relate to production impact and disease control costs. Economic losses, however, vary among regions and farms. JD results in reduced milk production, fertility issues, and eventual animal culling [48]. At herd level, economic costs relate to disease prevention, diagnosis, monitoring, animal replacement costs, reduced feed conversion ratio, susceptibility to comorbidities, and slaughter costs [28]. Importantly, increased incidence of clinical mastitis and/or subclinical mastitis may be associated with MAP infection in dairy cows [51], increasing disease treatment costs at cow and herd level. The economic impact of paratuberculosis at herd level is dependent on the herd size, infection rate, bacterial shedding, display of clinical symptoms, and cases of clinical disease [52]. Reduced milk yield is evident in subclinically infected cows and is more pronounced in clinically infected animals, with a reduction of 15 and 19.5%, respectively [28]. Farmers often cull animals with a low or decreased milk yield and replace the animal with a more efficient milk-producing cow, increasing replacement costs. Importantly, MAP is frequently introduced to dairy herds via the purchase of infected animals which are often asymptomatic [49], making culling and replacement less effective at controlling the disease. Additionally, fecal culture-positive cows have been culled for reproductive issues more frequently than culture-negative cows [53]. Challenges arise with calculating the economic impact of JD at herd and regional levels as subclinically infected animals are asymptomatic with clinical cases identified at late stages of disease.

At industry and consumer level, JD has an impact on price fluctuations of dairy products due to the elasticities of supply and demand [54]. Elimination of JD from dairy herds and subsequent increased dairy production may lead to a price decrease benefiting consumers, whereas proliferation of the disease reduces dairy production and increases prices at markets. As an externality, dairy production also affects those involved in the dairy food supply chain including producers, processors, distributors, and stakeholders. The economic impact of JD and dairy production on these businesses is also a factor which must be considered when assessing the overall economic losses caused by this infectious disease. The economic cost estimates for JD are typically less than for mastitis in dairy production, suggesting that JD may not represent a major financial concern for producers, which may contribute to a lack of monitoring of the economic costs relating to the disease [48].

### Control Measures

The financial burden, animal welfare, and possible zoonosis of JD are the major drivers for effective control measures. Indeed, there is increased concern globally relating to animal welfare and health in food production. Implementing biosecurity for infectious disease has two major elements: reducing the introduction of the infectious agent to the farm and reducing the likelihood of transmission within the herd. At present, there is no effective vaccine available for MAP which can be utilized, and the disease remains untreatable. Additionally, the current vaccine for MAP, which has questionable efficacy, compromises the diagnosis of bovine tuberculosis in cattle [32]. Prevention of disease at farm level and reducing vertical transmission are key areas where control needs to be implemented. The test-and-cull approach is the current method in use for disease prevention. This method, however, can miss subclinically infected animals and incurs replacement costs. Studies have shown that improving calf hygiene coupled with the test-and-cull approach is necessary to reduce herd prevalence, with reduced calf exposure the most significant measure [52]. Culling the offspring of infected cows may also be introduced as part of the control strategy as MAP in utero transmission is an important factor within herds [55]. Optimal hygiene and disease management is, therefore, essential to prevent horizontal transmission within herds. Protecting calves by preventing ingestion and reducing contact with MAP-loaded adult feces is also essential to reduce the incidence of disease [40]. Given the environmental robustness of MAP, pasture and grazing management can be applied to decrease transmission in extensively grazed livestock. Effective cleaning and sanitation of animal housing and milking parlors may reduce viable MAP numbers; however, the presence of organic matter can greatly reduce disinfectant efficacy. Further issues arise as nontuberculous *Mycobacteria* are resistant to chlorine-based disinfectants [56].

Low awareness of JD and poor implementation of biosecurity practices are issues present in the dairy industry. Producer awareness and education in disease prevention and control need to be improved relating to effective MAP control measures. The pathogenicity of MAP relates to its ability to generate specific phenotypes in different environments, leading to varying virulence characteristics. This genetic diversity makes developing effective vaccination programs difficult. Understanding this genetic diversity or plasticity is important to develop effective management protocols [50]. The interaction between livestock and wildlife reservoirs is another import aspect of infectious disease. There are numerous wildlife species that can transmit the pathogen to livestock farms, with leakage from farms to wildlife also an issue. Understanding the numerous risk factors associated with this contagious infectious pathogen will aid in reducing prevalence and is important in an effective biosecurity program. As a high level of producer participation is necessary for control programs to succeed, control programs are often hindered by a lack of farm participation [57], allowing trade of infected animals to occur. The World Trade Organization (WTO) states producers are required to take an “appropriate”, scientifically based level of sanitary measures to protect human and animal health. The World Organization for Animal Health (OIE), recognized by WTO, however, offers no guidance on paratuberculosis disease control [52]. Recommendations for trade of livestock relating to JD control are given by the International Association for Paratuberculosis in line with the Sanitary and Phytosanitary (SPS) Agreement of WTO [58].

Knowledge gaps exist in many areas which affect MAP control; differentiating clinical and subclinical disease by use of biomarkers, for example, may contribute to early detection [12]. Differentiating between MAP and other mycobacterial infections should improve the ability of ELISA tests to determine prevalence rates. At present, prolonged preclinical shedding phase, lack of test sensitivity, MAP environmental persistence, and poor management systems allow MAP to persist particularly in pastoral, seasonal dairy systems [44].

## 5. Conclusions

Johne’s disease is a major concern for animal health and food production industries as this wasting disease causes a significant economic impact globally. Disease management and control appear to be ineffective as prevalence rates remain high. The interaction between the infectious agent, the host, and the environment is diverse, making treatment of disease difficult and ineffective. To improve paratuberculosis control globally, active surveillance, producer awareness, and improved diagnostic testing and epidemiology are critical areas which must be focused on. The development of an effective vaccination program would greatly impact disease prevalence, however, such a preventative measure remains elusive due primarily to the ability of MAP to evade host immunity. Ingestion of MAP-contaminated milk by neonatal calves is a major route of transmission with evidence that infection can also occur across the placenta. Preventing animals from consuming MAP cells in colostrum and milk and decreasing contamination of the environment with feces or discarded milk are important aspects in preventing transmission. As disinfection biocides have limited efficacy against MAP, their use alone cannot be entirely relied on. Importantly, in dairy farming, farm personnel may not see the control of MAP as their priority, particularly as mastitis has such an economic impact on the industry. Farmers are the key participants for the successful implementation of an effective infectious disease control program in dairy herds.
